# High Diagnostic Utility Incorporating a Targeted Neurodegeneration Gene Panel With MRI Brain Diagnostic Algorithms in Patients With Young-Onset Cognitive Impairment With Leukodystrophy

**DOI:** 10.3389/fneur.2021.631407

**Published:** 2021-02-01

**Authors:** Zhiyong Chen, Yi Jayne Tan, Michelle M. Lian, Moses Tandiono, Jia Nee Foo, Weng Khong Lim, Nagaendran Kandiah, Eng-King Tan, Adeline S. L. Ng

**Affiliations:** ^1^Department of Neurology, National Neuroscience Institute, Tan Tock Seng Hospital, Singapore, Singapore; ^2^Lee Kong Chian School of Medicine, Nanyang Technological University, Singapore, Singapore; ^3^Human Genetics, Genome Institute of Singapore, A^*^STAR, Singapore, Singapore; ^4^Singhealth Duke-NUS Institute of Precision Medicine, Singapore, Singapore; ^5^Cancer & Stem Cell Biology Program, Duke-NUS Medical School, Singapore, Singapore; ^6^Neuroscience and Behavioural Disorders Program, Duke-NUS Medical School, Singapore, Singapore; ^7^Department of Neurology, National Neuroscience Institute, Singapore General Hospital, Singapore, Singapore

**Keywords:** NOTCH3, CADASIL, leukoencephalopathies, exome sequencing, cerebral small vessel disease, HtrA1 protein, human, Hereditary Diffuse Leukoencephalopathy with Spheroids

## Abstract

Leukodystrophies are a diverse group of genetic disorders that selectively involve the white matter of the brain and are a frequent cause of young-onset cognitive impairment. Genetic diagnosis is challenging. Data on the utility of incorporating brain magnetic resonance imaging (MRI) diagnostic algorithms with next-generation sequencing (NGS) for diagnosis in a real-life clinical setting is limited. We performed sequencing using a custom-designed panel of 200 neurodegeneration-associated genes on 45 patients with young-onset cognitive impairment with leukodystrophy, and classified them based on van der Knaap et al.'s MRI diagnostic algorithm. We found that 20/45 (44.4%) patients carried pathogenic variants or novel variants predicted to be pathogenic (one in *CSF1R*, two in *HTRA1* and 17 in *NOTCH3*). All patients with an established genetic diagnosis had an MRI brain pattern consistent with a specific genetic condition/s. More than half (19/37, 51.4%) of patients with MRI changes consistent with vascular cognitive impairment secondary to small vessel disease (VCI-SVD) had pathogenic variants, including all patients with pathogenic *NOTCH3* (17/19, 89.5%) and *HTRA1* variants (2/19, 11.5%). Amongst patients harboring pathogenic *NOTCH3* variants, 13/17 (76.5%) carried the p.R544C variant seen predominantly in East Asians. Anterior temporal white matter involvement was seen only in patients with pathogenic *NOTCH3* variants (6/17, 35.3%). Overall, we demonstrated a high diagnostic utility incorporating a targeted neurodegeneration gene panel and MRI-based diagnostic algorithms in young-onset cognitive impairment patients with leukodystrophy.

## Introduction

Leukodystrophies are genetically determined disorders that selectively involve the white matter of the brain. Etiologies are diverse, encompassing disorders caused by demyelination, hypomyelination, astrocytic and microglial defects of the white matter, making accurate diagnosis challenging.

Diagnostic algorithms based on magnetic resonance imaging (MRI) brain patterns have been proposed to aid in clinico-genetic diagnoses of leukodystrophies (1, 2). Next-generation sequencing (NGS) in the form of whole-exome sequencing (WES), as well as capture-based targeted panel sequencing have in recent years improved the diagnostic yield in patients with leukodystrophies and apparent sporadic leukoencephalopathies (3–6).

Data regarding the diagnostic utility of incorporating NGS and MRI brain diagnostic algorithms in the diagnosis of adult onset leukodystrophy in everyday clinical practice is limited. One study incorporating NGS and MRI brain diagnostic algorithms for the diagnosis of leukodystrophy found cases of non-concordance between the two diagnostic modalities. This is possibly related to the broad range of clinical presentations as well as the wide age-range of patients recruited to the study (4).

Here, we assess the diagnostic utility of incorporating a custom panel of 200 neurodegeneration-related genes (including genes related to dementia, Parkinson's disease and adult onset leukodystrophy) together with an MRI brain-based diagnostic algorithm published by van der Knaap et. al. (2) in the diagnostic workup of patients with young-onset cognitive impairment and leukodystrophy in a tertiary neurology referral center.

## Materials and Methods

### Patient Recruitment

Between January 2015 and July 2018, 577 patients with young-onset dementia [defined as 1. dementia occurring under the age of 65, 2. fulfilling the DSM-5 criteria for minor and major neurocognitive disorder (7)] were seen in the young onset dementia clinic of the National Neuroscience Institute, Singapore. Subjects with prominent white matter hyperintensities on T2 -weighted MRI of the brain that were symmetrical and met the inclusion criteria were recruited into the study.

Exclusion criteria included: Patients who declined recruitment into the study, acquired demyelinating disorders including inflammatory, infection, drug induced/toxic, neoplastic causes.

Information on clinical history and examination findings were collected during clinical encounters and through assessment of all available clinical records. All participants assessed by trained dementia specialists.

This study was approved by the Singapore Health Services Centralized Institutional Review Board (CIRB), and all participants provided informed consent. In patients with impaired decision-making capacity, consent was obtained from their legally acceptable representative.

### Neuroimaging

MRI sequences used for analysis included T1, T2, fluid attenuated inversion recovery (FLAIR), susceptibility weighted imaging (SWI) or gradient echo (GRE) sequence, and diffusion weighted imaging (DWI). Patients were classified qualitatively based on MRI brain diagnostic algorithms for leukodystrophies published by van der Knaap et al. (2). Briefly, classification was based on: (1). Confirmation of the presence of prominent symmetrical white matter abnormalities, features of which are suggestive of leukodystrophy; (2). if imaging is consistent with hypomyelination; (3). if imaging is consistent with other pathologies, whether lesions are multifocal or confluent; (4). for confluent lesions, the predominant localization of MRI abnormalities; and (5). whether there are special MRI characteristics. Patients with multifocal pattern of MRI changes were further subdivided into whether the changes were consistent with vasculopathy, and whether the vascular cognitive impairment was secondary to small vessel disease (VCI-SVD) or vasculopathy secondary to causes other than small vessel vasculopathy (VCI-non SVD). MRI brain diagnosis of VCI-SVD was made based on the STandards for ReportIng Vascular changes on nEuroimaging (STRIVE) recommendations (8). Quantitative assessment of white matter burden of periventricular and subcortical regions on MRI Brain imaging was made based on the modified Fazekas scale (9). Microbleeds were defined as homogeneous round signal loss lesions with a diameter of up to 5 mm on GRE, locations of microbleeds were categorized by cerebral region (cortico-subcortical, basal ganglia, brainstem, cerebellum).

### Targeted Neurodegeneration Gene Panel Sequencing

Whole blood was collected on study participants for genetic analysis. Genomic DNA was extracted from peripheral blood using QIAamp® DNA Blood Maxi Kit (Qiagen, Germany). Each sample was bar-coded and prepared for next-generation sequencing with the NEBNext Ultra II Library Prep kit (New England Biolabs). Twenty-nine genes related to adult onset leukodystrophy were sequenced as part of a panel of 200 neurodegenerative disease-related genes (full list of genes in Supplementary Table 1). Exonic sequences of these 200 genes (1.2 Mb) were captured with the NimbleGen SeqCap EZ choice <7 Mb (Roche) following the manufacturer's protocol. This panel was curated to target leukodystrophy genes that had been reported in literature to be associated with adult-onset leukodystrophy (1, 4, 6). We performed multiplexed capture in batches of 12 for a total of 576 samples which were sequenced on two lanes (288 samples per lane) of 151 bp paired-end sequencing with HiSeq4000 (Illumina). Sequence reads were aligned to the hg19 (build 37) human genome reference using BWA-MEM algorithm (BWA, v0.7.15) and SNP and indels were called using the Genome Analysis Tool-Kit (GATK, v3.7) Haplotype Caller following GATK best practices workflow (10–12). Variants were annotated using SIFT 4G (v2.4), PolyPhen-2 and ANNOVAR, and filtered for non-synonymous, frameshifts, stop-gain, splice-site and rare variants (MAF <5% in gnomAD population) (13–15). An average of 97.3% of the target sequences were covered with at least 15 reads across all 576 samples, with mean coverage of 114.5x across all targets per sample. In a subset of 44 patients diagnosed with vascular diseases, the mean coverage for the *NOTCH3* gene was 100.4x, with an average of 97.0% of *NOTCH3* targeted exons region (9,463 bp) covered by at least 15 reads (base quality ≥10, mapping quality ≥20). The mean coverage for the *HTRA1* gene was 96.2x, with an average of 73.9% of *HTRA1* targeted exons region (2,242 bp) covered by at least 15 reads (base quality ≥10, mapping quality ≥20), which is comparable with other exome sequencing studies (4, 6). Additionally, Sanger sequencing was performed across all nine *HTRA1* exons for all samples (including the patient sequenced by WES), and confirmed the presence of the reported *HTRA1* variants accordingly (Supplementary Table 2). The mean coverage for the *CSF1R* gene was 139.9x, with an average of 99.9% of *CSF1R* targeted exons region (5,164 bp) covered by at least 15 reads (base quality ≥10, mapping quality ≥20).

### Whole Exome Sequencing

One patient sample (Patient 19) was sequenced by WES. Whole exome was captured with the NimbleGen SeqCap EZ Human Exome v3.0 (Roche) following the manufacturer's protocol, and sequenced using HiSeq4000 with 150-bp paired end reads (Illumina). The same variant calling and annotation pipeline, as well as variant filtering criteria utilized for targeted panel sequencing reads was also conducted for the whole exome sequencing data. The mean coverage for *HTRA1* gene in this patient sample was 78.8x, with an average of 76.0% of *HTRA1* targeted exons region (2,049 bp) covered by at least 15 reads (base quality ≥10, mapping quality ≥20).

### Variant Prioritization

We prioritized the analysis of variants in leukodystrophy genes based on patient's MRI brain diagnosis. We removed synonymous variants and variants with minor allele frequency (MAF) > 0.01 on the gnomAD database. Identified variants were referenced against public databases including ClinVar (www.ncbi.nlm.nih.gov/clinvar/) and Varsome (https://varsome.com/). The pathogenicity of variants were classified based on the American College of Medical Genetics (ACMG) criteria (16). All pathogenic variants and novel variants predicted to be pathogenic were confirmed by Sanger sequencing (see details in Supplementary Material).

### Statistical Analysis

Statistical analyses were performed with IBM SPSS Statistics, version 23, IBM. Comparative analyses between patients with VCI-SVD with pathogenic *NOTCH3* variants and with VCI-SVD without identifiable pathogenic variants were performed using Mann-Whitney U test or 2-sample *t*-test as appropriate for continuous data, and with Fisher's exact test for categorical data. Two-tailed *p*-values of <0.05 were considered statistically significant.

## Results

**Figure 1:**
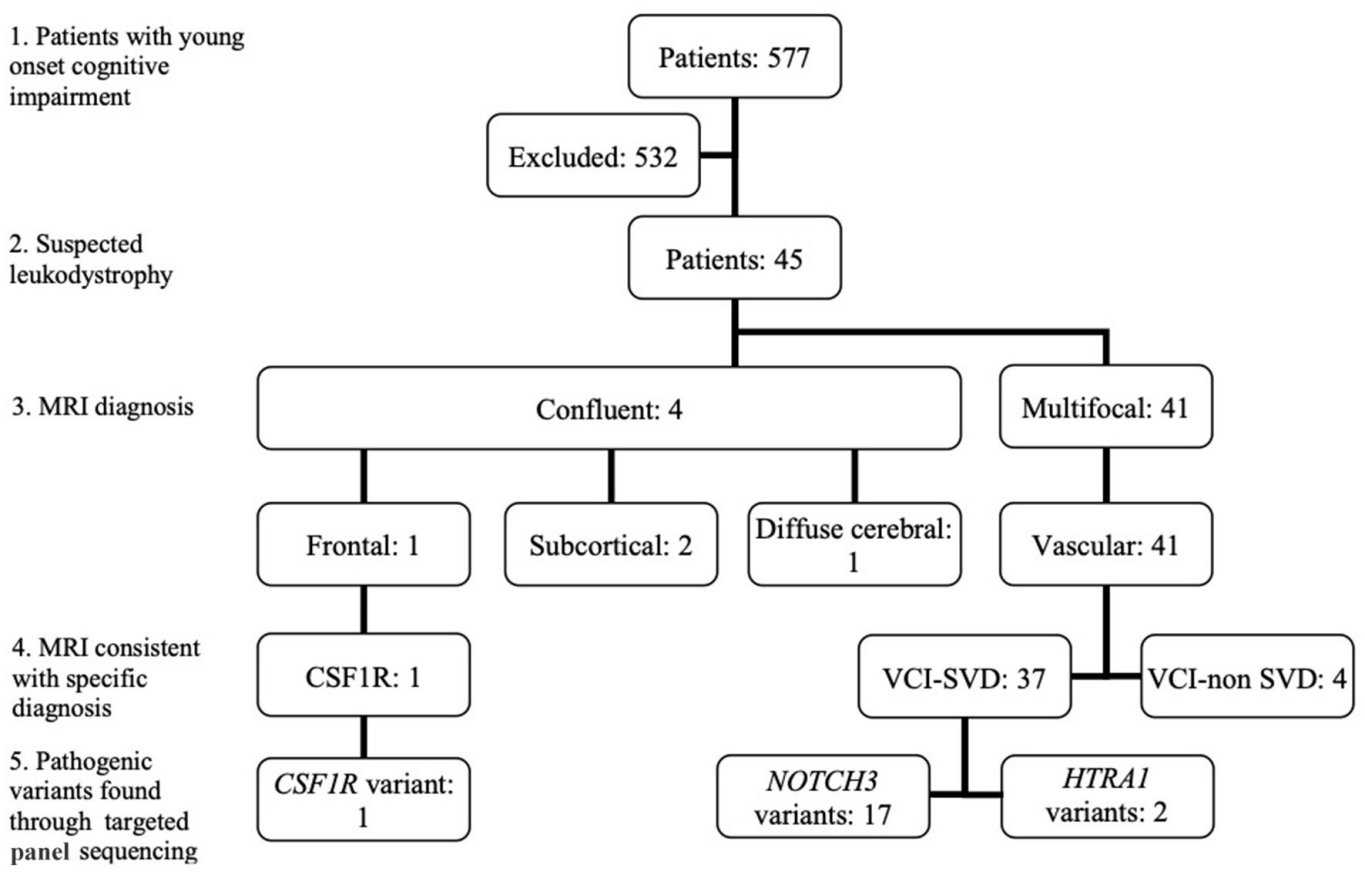
Classification of patients based on MRI brain diagnostic algorithm and pathogenic variants found. (1): Patients with young onset cognitive impairment; (2): Patients with suspected leukodystrophy on MRI Brain; (3): Classification of MRI brain findings based on van der Knapp et al.'s criteria; (4): Whether MRI brain was consistent with the diagnosis of a specific leukodystrophy; (5). Pathogenic variants found through targeted panel sequencing. CSF1R, CSF1R related leukoencephalopathy; VCI-SVD, vascular cognitive impairment secondary to small vessel disease; VCI-non SVD, vascular cognitive impairment not caused by small vessel disease.

Among 577 patients with young-onset dementia, 45 patients fulfilled the inclusion criteria (clinical demographics summarized in Table 1). The breakdown of patients based on MRI brain and genetic diagnosis is summarized in Figure 1. Based on van der Knapp's et al.'s MRI brain diagnostic algorithm, patients were classified as follows: multifocal (*n* = 41) and confluent (*n* = 4). Within the confluent group, frontal (*n* = 1), subcortical (*n* = 2), diffuse cerebral (*n* = 1).

**Table 1:** Demographics of patients with leukodystrophy on magnetic resonance imaging (MRI).

	**Patients (*N* = 45)**
Male (%)	24 (53.3)
Chinese (%)	40 (88.9)
Malay (%)	3 (6.7)
Filipino (%)	1 (2.2)
Japanese (%)	1 (2.2)
Median age at onset (range), years	54 (34–65)
Memory as first symptom (%)	20 (44.4)
Migraine with aura (%)	5 (11.1)
Haemorrhagic stroke (%)	6 (13.3)
Ischemic stroke (%)	21 (46.7)
Median MMSE (range)	25 (0–30)
Median MOCA (range)	22(0–29)
Depression (%)	22 (48.9)
Epilepsy (%)	4 (8.9)
Parkinsonism (%)	11 (24.4)
Hypertension (%)	30 (66.7)
Hyperlipidaemia (%)	25 (55.6)
Diabetes mellitus (%)	13 (28.9)
Smoking (%)	6 (13.3)
Alcohol (%)	2 (4.4)
Family History (%)	21 (46.7)

*MMSE, mini mental state examination; MOCA, Montreal cognitive assessment*.

**Figure 2:**
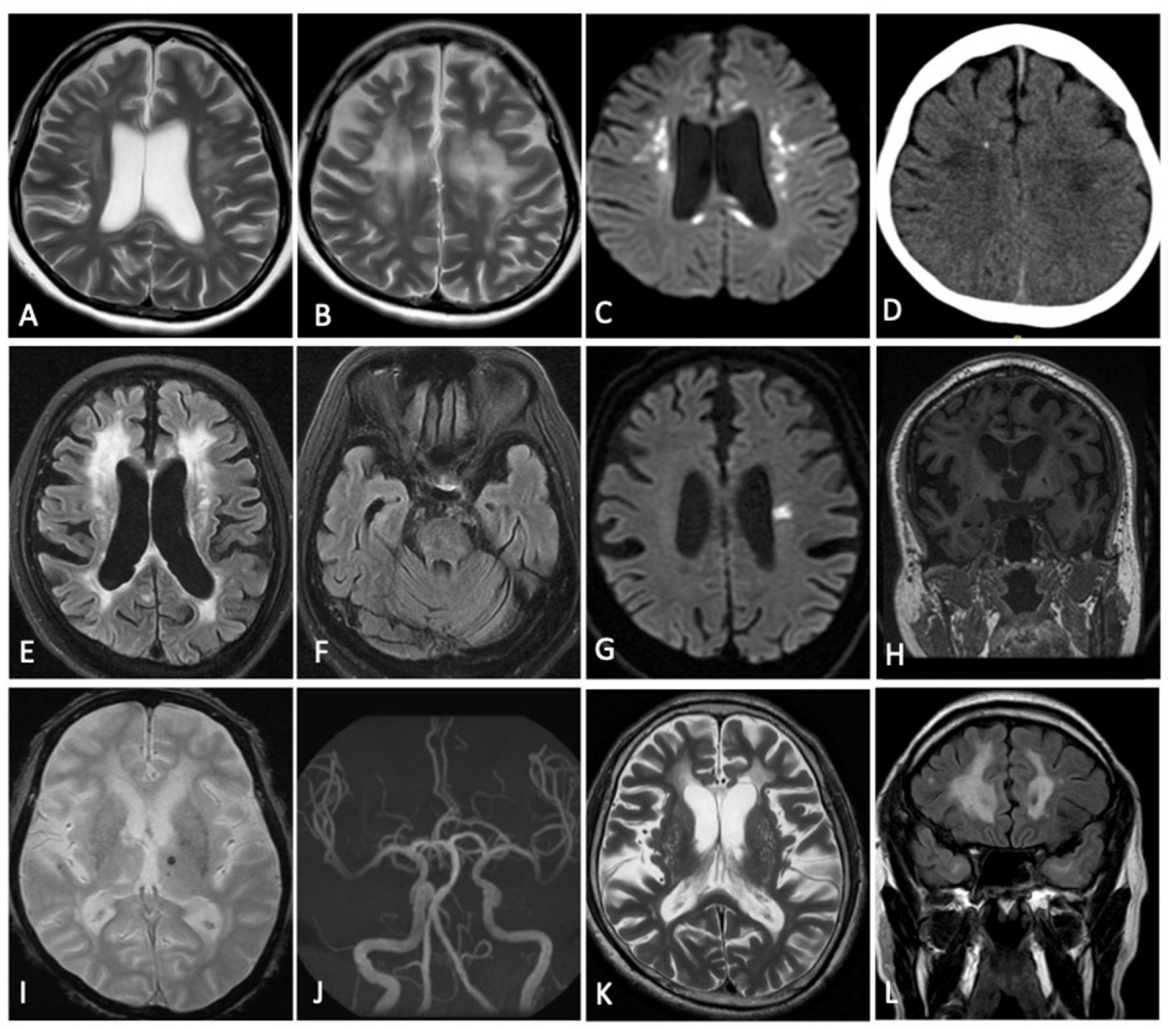
Imaging findings of patients with specific diagnoses. Patient with frontal predominant MRI pattern secondary to CSF1R-related leukoencephalopathy **(A–D)**: confluent frontal predominant white matter changes **(A,B)** associated with scattered areas of restricted diffusion **(C)** and an area of intraparenchymal calcification **(D)**. Patient with multifocal MRI pattern consistent with VCI-SVD secondary to heterozygous mutation of HTRA1 (CADASIL2) **(E–K)**: multifocal white matter hyperintensity **(E)** without involvement of the anterior temporal poles **(F)**, acute **(G)** and chronic **(H)** lacunar infarcts, microbleeds **(I)**, normal intracranial vasculature **(J)** with dilated perivascular spaces **(K)**. Patient with multifocal MRI pattern consistent with VCI-SVD secondary to heterozygous mutation of NOTCH3 (CADASIL1) **(L)**: multifocal white matter hyperintensity with involvement of the anterior temporal poles **(L)**. **(A,B,K)** Axial T2; **(C,G)** Axial DWI; **(D)** Axial CT Brain; **(E,F)** Axial T2 FLAIR; **(H)** Coronal T1; **(I)** Axial GRE; **(J)** MR Angiography; **(L)** Coronal T2 FLAIR.

The patient demonstrating a frontal-predominant MRI pattern had imaging features suggestive of *CSF1R-*related leukoencephalopathy (17), including periventricular calcifications seen on plain CT imaging that are highly suggestive of the disease (Figures 2A–D). Of the 41 patients in the multifocal pattern group, 37 had vascular cognitive impairment secondary to small vessel disease (VCI-SVD) (VCI-SVD, Figures 2E–L) while 4 had vascular cognitive impairment secondary etiologies other than small vessel disease (VCI-non SVD).

### Genetic Variants

Pathogenic variants or novel variants predicted to be pathogenic were identified in 20/45 cases (44.4%) (Figure 1, Table 2). Seventeen cases carried pathogenic *NOTCH3* variants, two had pathogenic variants in the *HTRA1* gene that is associated with *HTRA1*-cerebral small vessel disease, designated by OMIM as cerebral autosomal dominant arteriopathy with subcortical infarcts and leukoencephalopathy type 2 (CADASIL2, 616779), while one had a novel variant (p.T567M) in the colony stimulating factor 1 receptor (*CSF1R*) gene. More than half (19/37, 51.4%) of patients with MRI brain changes consistent with VCI-SVD had pathogenic variants, including all patients with pathogenic *NOTCH3* variants (17/19, 89.5%) as well as *HTRA1* variants (2/19, 11.5%).

**Table 2:** Clinical and MRI brain data of patients with pathogenic and likely pathogenic variants involving leukodystrophy-associated genes.

**Patient ID**	**Gender**	**Age at onset**	**Race**	**Symptom other than dementia**	**MRI pattern**	**Gene**	**Inheri-tance**	**Amino acid change**	**Polyphen-2 score**	**Polyphen-2 prediction**	**SIFT4G score**	**SIFT4G prediction**	**gnomAD freq (East Asians)**	**gnomAD freq (South Asians)**	**gnomAD freq (Non-Finnish Europeans)**	**ACMG criteria**
1.	F	34	Chinese	Parkinson	Frontal	*CSF1R*	AD	p.T567M	0.999	Damaging	0	Deleterious	0	0.00003	0.00003	Likely pathogenic
2.	F	43	Chinese	ICH	Multifocal SVD	*NOTCH3*	AD	p.R544C	0.462	Possibly damaging	0.042	Deleterious	0.0041	0.0001	0	Pathogenic
3.	F	44	Chinese	Stroke	Multifocal SVD	*NOTCH3*	AD	p.C134Y	1.0	Damaging	0	Deleterious	–	–	–	Likely pathogenic
4.	F	55	Chinese	Parkinson dystonia	Multifocal SVD	*NOTCH3*	AD	p.C1250R	1.0	Damaging	0	Deleterious	–	–	–	Likely pathogenic
5.	F	52	Chinese	Stroke ICH	Multifocal SVD	*NOTCH3*	AD	p.R544C	0.462	Possibly damaging	0.042	Deleterious	0.0041	0.0001	0	Pathogenic
6.	M	62	Chinese	ICH epilepsy	Multifocal SVD	*NOTCH3*	AD	p.R544C	0.462	Possibly damaging	0.042	Deleterious	0.0041	0.0001	0	Pathogenic
7.	M	42	Chinese	Stroke cerebellar	Multifocal SVD	*NOTCH3*	AD	p.R152C	0.846	Possibly damaging	0.035	Deleterious	–	–	–	Pathogenic
8.	M	56	Chinese		Multifocal SVD	*NOTCH3*	AD	p.R544C	0.462	Possibly damaging	0.042	Deleterious	0.0041	0.0001	0	Pathogenic
9.	M	63	Chinese	Stroke parkinson	Multifocal SVD	*NOTCH3*	AD	p.R587C	0.892	Damaging	0.102	Tolerated	0.0002	0	0	Likely pathogenic
10.	M	48	Chinese	Stroke parkinson	Multifocal SVD	*NOTCH3*	AD	p.R544C	0.462	Possibly damaging	0.042	Deleterious	0.0041	0.0001	0	Pathogenic
11.	M	64	Chinese	Stroke	Multifocal SVD	*NOTCH3*	AD	p.R544C	0.462	Possibly damaging	0.042	Deleterious	0.0041	0.0001	0	Pathogenic
12.	F	50	Chinese	Stroke	Multifocal non SVD	*NOTCH3*	AD	p.R544C	0.462	Possibly damaging	0.042	Deleterious	0.0041	0.0001	0	Pathogenic
13.	M	61	Chinese		Multifocal non SVD	*NOTCH3*	AD	p.R544C	0.462	Possibly damaging	0.042	Deleterious	0.0041	0.0001	0	Pathogenic
14.	F	63	Filipino		Multifocal SVD	*NOTCH3*	AD	p.R544C	0.462	Possibly damaging	0.042	Deleterious	0.0041	0.0001	0	Pathogenic
15.	F	53	Chinese	ICH psychiatric	Multifocal SVD	*NOTCH3*	AD	p.R544C	0.462	Possibly damaging	0.042	Deleterious	0.0041	0.0001	0	Pathogenic
16.	M	64	Chinese	Parkinson psychiatric	Multifocal SVD	*NOTCH3*	AD	p.R544C	0.462	Possibly damaging	0.042	Deleterious	0.0041	0.0001	0	Pathogenic
17.	F	43	Chinese	Stroke	Multifocal SVD	*NOTCH3*	AD	p.[R544C]; [R544C]	0.462	Possibly damaging	0.042	Deleterious	0.0041	0.0001	0	Pathogenic
18.	F	52	Chinese	Stroke	Multifocal SVD	*NOTCH3*	AD	p.[R544C]; [R607C]	0.462	Possibly damaging	0.042	Deleterious	0.0041; 0.00005	0.0001; 0	0; 0	Pathogenic; pathogenic
19.	M	41	Chinese	Stroke	Multifocal SVD	*HTRA1*	AD	p.P285L	0.995	Damaging	0.018	Deleterious	0.00005	0	0	Pathogenic
20.	M	59	Japanese	Stroke	Multifocal SVD	*HTRA1*	AD	p.R302*	NA	NA	NA	NA	0	0	0.000009	Pathogenic

*ACMG, American College of Medical Genetics; AD, autosomal dominant; ICH, intracranial hemorrhage; parkinson, parkinsonism; stroke, ischaemic stroke; SVD, small vessel disease*.

Of the 17 patients with pathogenic *NOTCH3* variants, 13/17 patients (76.5%) involved the c.R544C variant; 11 was heterozygous for p.R544C, one was homozygous for p.R544C, while one had a compound heterozygous p.R544C and p.R607C variant. All but one patient (92.3%) with variants involving the p.R544C variant were Chinese. We also identified a previously unreported heterozygous cysteine substituting p.C1250R variant (NM_000435: Exon 23, c.3748A>G). All previous cysteine affecting variants have been reported to be pathogenic. This variant had not previously been reported in the gnomAD database, and is predicted *in-silico* to be probably damaging (SIFT score 0.00, PolyPhen-2 score 1.00; Table 2). This variant is predicted to be likely pathogenic based on ACMG guidelines.

In two patients with VCI-SVD, we identified one patient with a heterozygous pathogenic missense p.P285L variant (NM_002775: c.854C>A) as well as one patient with a heterozygous pathogenic nonsense p.R302* variant (NM_002775.5: c.904C>T) in the HtrA serine peptidase 1 gene (*HTRA1*). Both variants is located in the serine protease domain and had been previously reported to be associated with *HTRA1*-cerebral small vessel disease /CADASIL2 (18–21).

In the patient with a frontal-based MRI pattern, a novel heterozygous missense variant in *CSF1R* (NM_001288705: c.C1700T, p.T567M) was identified. While this variant is located in the juxtamembrane domain instead of the tyrosine kinase domain (TKD) of *CSF1R*, and has not been reported amongst East Asians in the gnomAD database, and is predicted *in-silico* to be damaging (SIFT score 0.00, PolyPhen-2 score 0.99, Table 2), with *CSF1R* associated with a low rate of benign missense variation. This patient presented with a consistent clinical syndrome i.e., in her 30s with rapidly progressive parkinsonism and dementia. MRI showed frontal predominant white matter involvement associated with scattered areas of restricted diffusion on diffusion weighted imaging (DWI) and areas of intraparenchymal calcification on CT brain, features most consistent with adult-onset leukoencephalopathy with axonal spheroids (ALSP)/*CSF1R-*related leukoencephalopathy (Figures 2A–D). This variant is predicted to be likely pathogenic based on the ACMG guideline.

### Comparison Between VCI-SVD Patients With Pathogenic *NOTCH3* Variants Against VCI-SVD Patients Without Pathogenic Variants

Comparison was made between VCI-SVD patients with pathogenic *NOTCH3* variants with patients without pathogenic variants (Table 3). Anterior temporal white matter involvement was seen only in patients with pathogenic *NOTCH3* variants (6/17, 35.3% vs. 0/18, 0%; *p* = 0.008, Figure 2L). Basal ganglia hyperintensities were more frequently observed in patients with pathogenic *NOTCH3* variants than those without (16/17, 94.1% vs. 11/18, 61.1%; *p* = 0.041). There was no observable difference in the presence of hyperintensities in other brain regions as well as the presence of microbleeds between both groups. There were also no significant differences observed for other demographic and clinical parameters, including positive family history for stroke/dementia, and presence of cardiovascular risk factors in the form of hypertension, hyperlipidemia, diabetes mellitus and smoking.

**Table 3:** Comparison between VCI-SVD patients with pathogenic NOTCH3 variants with VCI-SVD patients without pathogenic variants.

	**NOTCH3 (*N* = 17)**	**Negative (*N* = 18)**	***P-*value**
Male (%)	7 (41.2)	8 (44.4)	1.000
Chinese (%)	16 (94.1)	15 (83.3)	0.603
Mean age at onset (range), years	54.0 (40–64)	55.9 (41–65)	0.344
First symptom: Cognitive impairment (%)	6 (35.3)	11 (61.1)	0.272
First symptom: Parkinsonism (%)	3 (17.6)	2 (11.1)	0.775
Migraine with aura (%)	1 (5.9)	3 (16.7)	0.603
Hemorrhagic stroke (%)	4 (23.5)	1 (5.6)	0.177
Ischemic stroke (%)	8 (47.1)	6 (33.3)	0.500
Mean MMSE (range)	25 (15–30)	22 (0–30)	0.273
Mean MOCA (range)	22 (9–28)	19 (0–29)	0.679
Depression (%)	7 (41.2)	4 (22.2)	0.237
Epilepsy (%)	1 (5.9)	1 (5.6)	1.000
Parkinsonism (%)	6 (35.3)	2 (11.1)	0.121
Tremor (%)	0 (0)	0 (11.1)	1.000
Rigidity (%)	6 (35.3)	2 (11.1)	0.121
Bradykinesia (%)	6 (35.3)	2 (11.1)	0.121
Gait difficulty (%)	4 (23.5)	2 (11.1)	0.506
Hypertension (%)	9 (52.9)	15 (83.3)	0.075
Hyperlipidaemia (%)	9 (52.9)	11 (61.1)	0.738
Diabetes mellitus (%)	1 (5.9)	6 (33.3)	0.088
Smoking (%)	1 (5.9)	2 (11.1)	1.000
Alcohol (%)	0 (0)	1 (5.6)	1.000
Family History (%)	10 (58.8)	6 (33.3)	0.181
MRI			
Mean Fazekas score WMH (range)	3 (1–3)	2.5 (1–3)	0.112
Mean Fazekas score PVH (range)	3 (1–3)	2 (0–3)	0.058
Anterior temporal (%)	6 (35.3)	0 (0)	0.008
External capsule (%)	13 (76.5)	9 (50.0)	0.164
PVH (%)	17 (100.0)	16 (88.9)	0.486
WMH (%)	17 (100.0)	18 (100.0)	1.000
BG hyperintensity (%)	16 (94.1)	11 (61.1)	0.041
Infratentorial hyperintensity (%)	11 (64.7)	9 (50.0)	0.500
Microbleeds overall (%)	10 (58.8)	6 (33.3)	0.181
Microbleeds cortical-subcortical (%)	8 (47.1)	5 (27.8)	0.305
Microbleeds BG (%)	10 (58.8)	6 (33.3)	0.181
Microbleeds brainstem (%)	9 (52.9)	4 (22.2)	0.086
Microbleeds cerebellum (%)	7 (41.2)	2 (11.1)	0.060

*BG, basal ganglia; MMSE, mini mental state examination; MOCA, Montreal cognitive assessment; PVH, periventricular hyperintensity; WMH, deep white matter hyperintensity*.

Features of parkinsonism were found in 6/17 (35.3%) of patients with pathogenic *NOTCH3* mutations, 3/17 (17.6%) of which presented with parkinsonism as the initial complaint. There were no observable differences in the features of parkinsonism between both groups.

## Discussion

We incorporated van der Knaap et al.'s MRI brain diagnostic algorithm together with sequencing using a targeted neurodegeneration gene panel to analyze 45 young-onset cognitive impaired patients with leukodystrophy, achieving a diagnostic yield of 44.4%.

All patients with an established genetic diagnosis had an MRI brain pattern consistent with a specific genetic condition/s. This included 19/37 (51.4%) of patients with VCI-SVD, of which 17 carried pathogenic *NOTCH3* variants, and two carried pathogenic heterozygous *HTRA1* variants. We also identified a patient with MRI brain consistent with CSF1R-related leukoencephalopathy who harbored a novel p.T567M variant of the *CSF1R* gene.

Our findings run counter to that of a previous study by Kunii *et al*. that found cases of non-concordance between MRI brain and genetic diagnoses (4). This is potentially due to the restriction of our cohort to patients with young-onset dementia, leading to a patient group with more homogenous baseline characteristics, as opposed to Kunii's heterogenous cohort which consisted patients of a broad age range. As demonstrated by our study, the MRI brain diagnostic algorithm has the added purpose of phenotypic resolution of novel variants of unknown significance (VUS) in accordance to American College of Medical Genetics (ACMG) guidelines. The incorporation of an MRI brain diagnostic algorithm together with targeted panel sequencing is therefore a reliable methodology with high diagnostic utility in a real-life clinical setting.

In our study, we did not identify pathogenic mutations in neurodegeneration related genes associated with dementia, Parkinson disease as well as loci/SNPs reported in stroke-related genome-wide association studies (GWAS). While mutations in some genes associated with Alzheimer's disease, frontotemporal dementia and young-onset Parkinson disease have been associated with increased white matter hyperintensity (22–24) on MRI brain, our study demonstrates that in cases well selected to fit the diagnosis of leukodystrophy, further screening for these neurodegeneration related genes does not appear to increase diagnostic yield.

We found that anterior temporal white matter involvement appears to be less frequent in our *NOTCH3* positive patients as compared to Western and Japanese patients. Similar, anterior temporal involvement was only observed in 44% in both the Taiwanese as well as a Korean cohorts (25, 26). This could be explained by the high frequency of the p.R544C variant, which is located on exon 11, within our patients with pathogenic *NOTCH3* mutations. This variant also accounts for 70.5 and 90.3% of Taiwanese and Korean CADASIL patients, respectively (25, 27). Pathogenic variants involving exons 3 and 4 are more commonly observed in Western and Japanese CADASIL patients (28–30). The presence of anterior temporal involvement non-etheless appears to be a useful diagnostic feature for CADASIL within our cohort.

We found that 6/17 (35.3%) of *NOTCH3* positive patients in our cohort had features of parkinsonism, while 3/17 (17.6%) presented with parkinsonism as the first symptom. This is in contrast to Western cohorts, whereby parkinsonism and gait disturbances were described as late features of CADASIL (28, 31). While the prevalence of parkinsonism in the Taiwanese CADASIL cohort was not explicitly reported, gait difficulties had been similarly reported in 13.8 and 16.8% as an initial symptom and throughout the disease course, respectively. Further studies may be necessary to further evaluate genotype-phenotype correlations between different *NOTCH3* variants.

Additionally, we found that patients with pathogenic NOTCH3 variants demonstrated a greater burden of basal ganglia T2 hyperintensities compared to patients without pathogenic *NOTCH3* variants. Even though there was no statistical difference, there was a trend toward increased frequency of the presence of T2 hyperintensities in other brain regions as well as the presence of microbleeds in patients with pathogenic *NOTCH3* variants as opposed to patients without. These features are consistent with the diffuse intracerebral small vessel arteriopathy that underlies the pathogenesis of CADASIL, and is consistent with findings seen in other studies of patients with CADASIL (32).

Additionally, there are no significant differences in terms of onset of first symptom, frequency of family history of dementia as well as the other traditional cardiovascular risk factors of hypertension, hyperlipidemia, diabetes mellitus and cigarette smoking between VCI-SVD patients with pathogenic *NOTCH3* variants and VCI-SVD patients without identified variants. Given that more than half of VCI-SVD patients had identified pathogenic variants, the presence of concomitant cardiovascular risk factors or lack of family history should therefore not deter the physician from considering genetic testing in a patient with leukodystrophy with imaging findings suggestive of VCI-SVD. Conversely, given that nearly 60% of patients with pathogenic *NOTCH3* variants reported a positive family history of dementia, family history should be actively elicited during the course of the diagnostic evaluation as its presence increases the index of suspicion of an underlying genetic disorder.

Our study identified heterozygous pathogenic variants in the *HTRA1* gene associated with the recently reported CADASIL2 to be the second most common genetic cause of VCI-SVD within our cohort, constituting 11.5% of patients with VCI-SVD with identified pathogenic variants. While it had been shown that heterozygous *HTRA1* variants make up the second largest proportion of VCI-SVD patients in Taiwanese Han-Chinese, Japanese, Italian and French cohorts (18, 19, 33, 34), similar findings have so far not been reported in other leukodystrophy genetic sequencing studies. This demonstrates the rapidly progressing diagnostic yield of genetic testing in leukodystrophy, it also highlights once again the clinical utility of MRI brain diagnostic algorithm.

Our study identified a novel p.T567M variant occurring in the juxtamembrane domain rather than the tyrosine kinase domain (TKD) of the *CSF1R* gene in a patient with a clinical phenotype consistent with ALSP/*CSF1R-*related leukoencephalopathy, classified as likely pathogenic based of ACMG criteria. Currently, more than 80 pathogenic/likely pathogenic *CSF1R* variants have been reported, almost all of which are located in the TKD of the protein (17).

In terms of cost economies, the lowest price of an NGS panel of 400 genes for the evaluation of leukodystrophy in Singapore is S$600 (USD$450), which corresponded to an average cost of USD$1.10 per gene. Considering that traditional Sanger sequencing is priced around USD$200–400 per gene, the utilization of NGS panels encompassing either all leukodystrophy genes or for genes associated with a specific MRI brain pattern appears to be more effective cost wise as well as diagnostically.

We were unable to identify pathogenic variants in 26/45 (57.8%) patients. This included seven patients (two subcortical, one diffuse cerebral, 4 VCI-non SVD), for which MRI brain pattern was not consistent with a specific genetic condition/s, as well as 18 patients for which MRI brain pattern was consistent with VCI-SVD. This underlines the limitation of panel sequencing, which unlike WES or WGS, does not allow for future re-analysis of DNA to detect for mutations in leukodystrophy genes that are discovered after initial panel curation (in this case the *CLCN2, AARS, CTSA*, and *RNF216* genes).

However, for patients with a clear MRI brain pattern, panel sequencing continues to be a feasible option due to its greater ease of analysis, greater depth of coverage as well as its greater affordability as compared to WES or WGS. For patients whose MRI brain patterns are not consistent with a clear genetic condition, we suggest that WES or WGS be performed instead.

In conclusion, we found a high clinical utility of incorporating targeted panel sequencing and MRI brain diagnostic algorithms in young-onset patients with cognitive impairment with leukodystrophy, achieving a diagnostic yield of 44.4%.

## Data Availability Statement

The original contributions presented in the study are included in the article/Supplementary Material, further inquiries can be directed to the corresponding author/s.

## Ethics Statement

The studies involving human participants were reviewed and approved by Singhealth CIRB. The patients/participants provided their written informed consent to participate in this study.

## Author Contributions

ZC, AN, and E-KT: Drafting or revising the manuscript for intellectual content. AN and NK: Study design. YT, ML, and MT: Acquisition of data. ZC, JF, WL, and AN: Analysis and interpretation of the data. All authors approved the final version of the manuscript.

## Conflict of Interest

The authors declare that the research was conducted in the absence of any commercial or financial relationships that could be construed as a potential conflict of interest.

## Abbreviations

ALSP: adult-onset leukoencephalopathy with axonal spheroids and pigmented glia
CADASIL: Cerebral autosomal dominant arteriopathy with subcortical infarcts and leukoencephalopathy
CI: cognitive impairment
CSF1R: CSF1R related leukoencephalopathy
CVSD: cerebral small vessel disease
FLAIR: fluid attenuated inversion recovery
GRE: gradient echo
GWAS: genome-wide association studies
MRI: magnetic resonance imaging
NGS: next-generation sequencing
OMIM: online Mendelian inheritance in man
SWI: susceptibility weighted imaging
TKD: tyrosine kinase domain
VCI-non SVD: vascular cognitive impairment not caused by small vessel disease
VCI-SVD: vascular cognitive impairment secondary to small vessel disease
VUS: variants of unknown significance
WES: whole exome sequencing.
