# Supplementary material for: High Diagnostic Utility Incorporating a Targeted Neurodegeneration Gene Panel With MRI Brain Diagnostic Algorithms in Patients With Young-Onset Cognitive Impairment With Leukodystrophy
Source: Front Neurol. 2021 Feb 1;12:631407. doi: 10.3389/fneur.2021.631407 (PMC7882677; doi:10.3389/fneur.2021.631407)
Supplement: Supplementary file 1 [file Table_1.docx]

**Supplementary Materials and Methods**

**Sanger sequencing**

After amplification with exTEN 2x PCR Master Mix (1^st^ Base) and primers listed in Table 2, PCR products were treated with FastAP Thermosensitive Alkaline Phosphatase (Thermo Scientific) and Exonuclease I (Thermo Scientific) and subjected to sequencing following standard protocol from BigDye® Terminator v3.1 Cycle Sequencing Kit (Applied Biosystems). The amplified products were purified by magnetic beads clean up kit before loading into ABI 3730xl DNA Analyzer for DNA sequencing.

**Table 1. List of 200 neurodegenerative disease-related genes.**

| **Alzheimer’s disease, Frontotemporal dementia, vascular dementia and inherited vasculopathy-related genes (n=127)** | | | | | | | | | |
| --- | --- | --- | --- | --- | --- | --- | --- | --- | --- |
| ABCA7 | ABCD1 | ACE | ACOX1 | ADAM10 | APOC1 | APOE | APP | ARSA | ARID5B |
| ASPA | KCTD2 | AUH | ATXN2 | BIN1 | C11orf30 | C9ORF72 | CALHM1 | CASS4 | CD2AP |
| CD33 | CELF1 | CHCHD10 | CHMP2B | CHRNA9 | CLU | CNTNAP2 | COL4A2 | COL4A1 | CR1 |
| CTC1 | CSF1R | CYP11B2 | CYP19A1 | CYP27A1 | CYP2D6B | CYP2J2 | DARS2 | DNMT1 | DSG2 |
| DYRK1A | DYSF | ECHDC3 | EIF2B1 | EIF2B2 | EIF2B3 | EIF2B4 | EIF2B5 | EPHA1 | ERCC6 |
| ERCC8 | EPHA4 | FBF1 | FGB | FERMT2 | FUS | GAB2 | GALC | GBE1 | GCDH |
| GFAP | GLA | GRN | GSK3B | HEXA | HLA-DRA | HLA-DRB1 | HLA-DRB5 | HS3ST1 | HTRA1 |
| IL23R | INPP5D | L2HGDH | LMNB1 | LRRTM3 | MADD | MAN2B1 | MAPT | MEF2C | MLC1 |
| MRPL38 | MS4A4A | MTHFR | NCAM2 | NEDD9 | NLRP3 | NME8 | NOTCH3 | NXPH1 | NYAP1 |
| OPTN | SERPINE1 | PAXIP1 | PICALM | PLP1 | PLD3 | PRNP | PSAP | PSEN1 | PSEN2 |
| PTK2B | RAB38 | REST | RIN3 | RUNX1 | SIRT2 | SLC17A5 | SLC2A14 | SORL1 | SQSTM1 |
| TARDBP | TBK1 | TMEM106B | TOMM40 | TP63 | TRIM47 | TRIM65 | TREM2 | TREX1 | TYMP |
| TRIP4 | TYROBP | UNC5C | UPP2 | VCP | WBP2 | ZCWPW1 |  |  |  |
| **PD related genes (n=73)** | | | | | | | | | |
| ACMSD | APOOP2 | ATP13A2 | BCKDK | BST1 | CCDC62 | CHCHD2 | COQ2 | CRAT | DDRGK1 |
| DGKQ | DLG2 | DNAJC13 | DNAJC5 | DNAJC6 | EIF4G1 | FAM186A | FAM47E | FBXO7 | FGF20 |
| GAK | GBA | GCH1 | GPNMB | GUCY1A3 | HIP1R | HLA-DRA | INPP5F | ITGA8 | ITPKB |
| KANSL1 | KRT8P25 | LAMC2 | LAMP3 | LRRK2 | MC1R | MCCC1 | MIR4697 | MMP16 | NMD3 |
| NSF | NUCKS1 | PARK2 | PARK7 | PINK1 | PLEKHM1 | PM20D1 | RAB25 | RAB7A | RAB29 |
| RAB7B | RAI1 | RIT2 | SCARB2 | SIPA1L2 | SLC2A13 | SLC41A1 | SLC45A3 | SLC7A4 | SMPD1 |
| SNCA | SPPL2B | SREBF1 | STBD1 | STK39 | STX1B | SYT11 | TMEM163 | TMEM175 | TMEM229B |
| USP25 | VPS13C | VPS35 |  |  |  |  |  |  |  |

200 neurodegenerative disease-related genes were selected for a custom commercial exome panel, which included genes selected from GWAS studies, risk variants as well as causal genes related to Alzheimer’s disease, Frontotemporal dementia, inherited vasculopathies as well as Parkinson’s disease.

**Table 2. Primers used for sequencing exons of *HTRA1*.**

| Target |  | Primer Sequence |
| --- | --- | --- |
| Exon 1 | F | 5’- GAAGGACGCGAATCTCAGCGAGAG -3’ |
|  | R | 5’- CCGCAGAAGCAGGTCCGAGCTGG -3’ |
| Exon 2, 3 | F | 5’- GGCTTCCTCTAACCCATGTC -3’ |
|  | R | 5’- CGAGAATCAGCTGCAATCAG -3’ |
| Exon 4, 5 | F | 5’- CCTGCTTGGTTTTCCATGAT -3’ |
|  | R | 5’- CCCTGCCTCAAAAACAAACA -3’ |
| Exon 6 | F | 5’- AGCTCAGGGACTTCTTTCAGG -3’ |
|  | R | 5’- GCTTTTCCCTTCCCCAAGT -3’ |
| Exon 7 | F | 5’- GTACCCTTCTGTGGCCCTTC -3’ |
|  | R | 5’- CCCGTGAGCACCTTCTGA -3’ |
| Exon 8 | F | 5’- GGGAACTGGTGAGAGCTGAG -3’ |
|  | R | 5’- CACGTCTGTCAAGGATCAACA -3’ |
| Exon 9 | F | 5’- CAGACCAGGAGGAATGGAAA -3’ |
|  | R | 5’- AACAAGCTGCTCTTGGGAAA -3' |
